# Pathogenicity and Genomic Features of a DAdV‐3 Strain Causing Severe Hepatitis in Muscovy Ducklings

**DOI:** 10.1155/tbed/7156678

**Published:** 2026-10-08

**Authors:** Chunhua Zhu, Zhiwang Tang, Pei Yang, Longfei Chen, Guanghua Fu, Qizhang Liang, Zhen Chen, Cuiteng Chen, Yu Huang, Chunhe Wan

**Affiliations:** ^1^ Fujian Provincial Key Laboratory for Avian Diseases Control and Prevention Institute of Animal Husbandry and Veterinary Medicine Fujian Academy of Agricultural Sciences Fuzhou 350013 China faas.cn

**Keywords:** duck adenovirus type 3, genetic characterization, hepatocyte injury, pathogenicity, virus isolation

## Abstract

Over the past decade, there has been an increase in reports of duck adenovirus type 3 (DAdV‐3) infections in Muscovy ducks across various provinces in China, with mortality rates reaching as high as 43%. The rising incidence of this disease has led to significant economic losses for the waterfowl farming industry in China. In this study, a DAdV‐3 strain was isolated from a Muscovy duck farm in Fujian province, which we designated as the FJNP‐2021 strain, and further explored its pathogenicity and genetic characteristics. Pathogenicity analysis demonstrated that this FJNP‐2021 strain induces characteristic pathological changes in Muscovy ducklings, exhibiting a broad tissue tropism, with the liver exhibiting the most severe lesions. The ultrastructural analysis revealed significant accumulation of lipid droplets and structurally disordered endoplasmic reticulum (ER) surrounding mitochondria in the hepatocytes of Muscovy ducklings postinfection. Moreover, in vivo DAdV‐3 infection significantly downregulated ferroptosis suppressive genes (*SLC7A11*, *GPX4*, and *FTH1*) while upregulating proferroptotic genes *TP53* and *CHAC1*. Our study provides dual ultrastructural morphology and molecular evidence that DAdV‐3–induced hepatic lipid metabolism disorder triggers ferroptosis, thereby exacerbating hepatocyte injury. Phylogenetic analysis revealed that the complete genome sequence of the FJNP‐2021 strain exhibited 92.3% identity with the representative strain GR from France, which was clustered in a different evolutionary lineage. Furthermore, the FJNP‐2021 strain was grouped within the same lineage as domestic DAdV‐3 strains, consistent with the genetic characteristics of the predominant epidemic strains of DAdV‐3 in China. Notably, the Fiber1 genes of the Chinese DAdV‐3 isolates formed two distinct subclades, and the Fiber1 gene of the FJNP‐2021 strain clustered within the major subclade. In conclusion, this study analyzed the pathogenicity and genetic characteristics of all available DAdV‐3 strains, enriching our understanding of the genomic diversity of Chinese isolates from 2014 to 2024 and providing a basis for further studies on DAdV‐3 evolution and pathogenesis.

## 1. Introduction

Fowl adenovirus (FAdV) is widely distributed worldwide [1]. According to the latest report from the International Committee on Taxonomy of Viruses (ICTV), the family *Adenoviridae* is classified into six genera: *Mastadenovirus*, *Aviadenovirus*, *Atadenovirus*, *Ichtadenovirus*, *Siadenovirus*, and *Testadenovirus* [2]. FAdV is characterized as a nonenveloped icosahedral virus particle, with its entire genome encapsulated by capsid proteins. Viral proteins can influence the viral replication cycle through their interactions with various host factors [3, 4]. These proteins are categorized into structural and nonstructural proteins based on their distinct functions, with structural proteins primarily consisting of capsid and core proteins. Capsid proteins play a crucial role in the interactions among viral proteins. The main capsid proteins include Hexon, Penton, and Fiber. Research has confirmed that Hexon and Fiber proteins are significant structural components associated with virulence and the host’s immune response to FAdVs [5]. Hexon, the predominant protein constituting the FAdV capsid, contains specific antigenic determinants, as well as minor species‐specific antigenic determinants, that serve as targets for neutralizing antibodies [6]. Fiber1 mediates the attachment of viruses to host cell receptors via its knob domain, thereby initiating the infection process [7, 8]. In contrast, Fiber2 acts as a primary virulence determinant, affecting the efficiency of viral replication and pathogenicity, and is currently the preferred target for subunit vaccine development against FAdV [9].

Duck adenovirus (DAdV) consists of viral members from two genera: DAdV type A (DAdV‐A) of the genus *Barthadenovirus* (formerly *Atadenovirus*) [10] and *Aviadenovirus anatis* of the genus *Aviadenovirus*, which includes DAdV‐2 [11] and DAdV type 3 (DAdV‐3) [12, 13], *Aviadenovirus cairinae* (DAdV‐4) [14, 15], *Aviadenovirus anatidae* (DAdV‐5), and *Aviadenovirus cerasi* (DAdV‐6), as well as the duck‐origin *Aviadenovirus hydropericardii* (FAdV‐4) [16, 17]. Domestically, DAdV‐A and DAdV‐2 (also referred to as DAdV‐3 or DAdV‐B) have been relatively well‐reported, while reports on DAdV‐4 and duck‐origin FAdV‐4 remain sporadic [14, 18, 19]. DAdV‐A, also known as DAdV type 1 (DAdV‐1) or egg drop syndrome virus (EDSV), is responsible for decreased egg production and compromised egg quality in various avian species, including chickens, ducks, geese, and quails [20]. The first case of DAdV‐2 infection in Muscovy ducks was reported in France, exhibiting clinical symptoms such as emaciation and unsteady standing [11]. Bouquet et al. [21] confirmed through neutralization tests that this virus was distinct from previously known members of the genus *Aviadenovirus*, representing a novel adenovirus. In 2014, Marek et al. [11] obtained the complete genome sequence of DAdV‐2 utilizing high‐throughput sequencing technology. That same year, Zhang et al. [13] reported a DAdV‐2 variant infection associated with acute hepatitis in Guangdong, China. The representative isolate CH‐GD‐12‐2014 (GenBank ID: KR135164) exhibited a 91.7% nucleotide identity with the DAdV‐2 GR strain, yet it was not identical to the earlier DAdV‐2 GR strain. A novel DAdV strain, CH‐GD‐12‐2014, isolated from Chinese Muscovy ducks in 2014, was found to possess two fiber genes (Fiber1 and Fiber2), in contrast to the DAdV‐2 GR strain, which contained only a single fiber gene, leading to its classification as DAdV‐3.

Following DAdV‐3 infection, Muscovy ducks exhibited necropsy lesions characterized by liver and kidney swelling, as well as hemorrhage. The morbidity rate ranged from 40% to 55%, while mortality varied between 35% and 43% [22]. Currently, the clinical incidence of DAdV‐3 is increasing. Furthermore, DAdV‐3 not only affects Muscovy ducks but also shelducks [22], indicating a potential risk for cross‐host transmission that poses significant threats to the waterfowl industry [22–24]. Research on DAdV‐3 has primarily focused on epidemiological investigation [25], virus isolation, and the development and application of detection methods [26–28], with relatively few studies addressing its infection and pathogenic mechanisms. Our laboratory previously collected liver tissues from diseased Muscovy ducks in Nanping, Fujian Province, and identified the isolated virus as DAdV‐3. This study aims to investigate the biological characteristics, pathogenicity, and genomic features, thereby providing a theoretical foundation for the further exploration of its pathogenic mechanisms.

## 2. Materials and Methods

### 2.1. Strains and Muscovy Ducks

DAdV‐3 was isolated from a Muscovy duck farm in Nanping, Fujian Province. Muscovy duck embryos and ducks were purchased from the breeding duck farm of Zhangzhou Changlong Agriculture and Animal Husbandry Co., Ltd. Healthy duck embryos were used to extract kidney tissues for the preparation of primary duck embryo kidney (DEK) cells. Liver tissues from the embryos were simultaneously collected for PCR testing to detect DAdV‐3 infection. Prior to the challenge experiment, serum and swab samples from the Muscovy ducks were collected. Serum neutralization tests and PCR confirmed that all samples were negative for DAdV‐3 and free from infections by pathogens such as FAdV‐4, duck circovirus, duck hepatitis A virus type 3, novel Muscovy duck parvovirus (MDPV), duck hepatitis B virus (DHBV), adeno‐associated virus (AAV), and H9 subtype avian influenza virus.

### 2.2. DAdV‐3 Strain Culture

Under aseptic conditions, kidney tissues from ~30‐day‐old duck embryos were excised, washed three times in DMEM containing 1% penicillin/streptomycin, and transferred to a new petri dish. Surgical scissors were used to mince the kidney tissues into small pieces. An equal volume of trypsin was added, and the mixture was incubated at 37°C for digestion. Each digestion lasted ~2 min and was repeated five times. The tissue suspension was thoroughly pipetted to obtain a single‐cell suspension. After centrifugation at 300 × g for 5 min, the supernatant was discarded, and the cell pellet was collected. The primary DEK cells were then resuspended in a medium to a concentration of 1 × 10^6^ cells/mL and transferred to cell culture flasks for cultivation.

When the DEK cells have grown to a fresh monolayer, they were inoculated with DAdV‐3 FJNP‐2021 strain solution at a concentration of 200 TCID_50_, utilizing DMEM medium supplemented with 2% fetal bovine serum (FBS) (OriCell, Australia) and 1% penicillin/streptomycin (Gibco, New York, USA). Prior to infection, the cells were washed three times with DMEM medium containing 1% penicillin/streptomycin to eliminate serum and dead cells. Subsequently, 4 mL of the virus solution was added to a T75 cell culture flask. The flask was incubated in a 37°C, 5% CO_2_ incubator for 2 h. After incubation, the virus solution was discarded, and it was replaced with DMEM medium containing 2% FBS and 1% penicillin/streptomycin for maintenance culture. The cytopathic effects were observed daily, and DAdV‐3 was harvested when ~80% cytopathic effect is observed around 4 days postinoculation. We continue serial passage until DAdV‐3 stably proliferates for eight generations in the DEK cells and measure its viral titer TCID_50_.

### 2.3. Ultrastructural Analysis Post‐DAdV‐3 Infection

The DEK cell suspension was adjusted to a concentration of 1.0 × 10^6^ cells/mL and seeded into culture flasks for overnight incubation to form a cell monolayer. The experimental group was inoculated with DAdV‐3, whereas uninfected normal cells were set as the control group. The infected primary kidney cells were gently rinsed twice with phosphate‐buffered saline (PBS) post‐DAdV‐3 infection at 48 h. 0.25% Trypsin‐EDTA (Gibco, New York, USA) was then added to digest the DEK cells for 3 min. After digestion, the cells were centrifuged at 300 × g for 5 min at room temperature, and the cell pellet was then resuspended in 500 μL of 2.5% glutaraldehyde solution and fixed overnight at 4°C. After fixation, the cells were pelleted by low‐speed centrifugation, and the supernatant was discarded. The cell pellet was then pre‐embedded in 1.5% agarose and incubated overnight at 4°C. The samples were washed three times with PBS for 15 min each to thoroughly remove residual reagents. The samples were immersed in 1% osmium tetroxide solution for postfixation for 1.5 h. Following this step, the samples were dehydrated with a graded ethanol series, embedded in epoxy resin, and cut into ultrathin sections. The ultrathin sections were stained with 3% uranyl acetate and lead citrate. Finally, the prepared ultrathin sections were observed using a transmission electron microscope (Hitachi HT7700, Tokyo, Japan).

Fresh liver tissues were harvested immediately from moribund ducklings. The liver tissues were rapidly cut into small tissue cubes of ~1 mm^3^. The tissue blocks were promptly immersed in precooled 2.5% glutaraldehyde solution and fixed overnight at 4°C. The samples were washed three times with PBS for 15 min each to thoroughly remove residual reagents. The samples were immersed in 1% osmium tetroxide solution for 1.5 h of postfixation. The ultrathin sections were stained with 3% uranyl acetate and lead citrate. Finally, the prepared ultrathin sections were observed using a transmission electron microscope (Hitachi HT7700, Tokyo, Japan).

### 2.4. Viral DNA Extraction

The prepared DEK cells were diluted to a density of 1 × 10^6^ cells/mL and plated into six‐well plates. The virus solution was diluted at a ratio of 1:1000 and subsequently added to the six‐well plates, alongside a control group that was not inoculated with the virus. DAdV‐3 samples were collected at 6, 12, 24, 48, 72, 96, and 120 h postinfection (hpi) of the primary kidney cells. For DNA extraction, 1 mL of the DAdV‐3 virus suspension was mixed with two 3 mm magnetic beads and homogenized at 50 Hz for 80 s in a tissue homogenizer (Sakezi, Anhui, China) set at 10°C. Subsequently, 200 μL of the supernatant was used to extract sample DNA following the protocol of the Biospin Viral DNA Extraction Kit (Hangzhou Bioer Technology Co., Ltd., Zhejiang, China), resulting in the yield of viral genomic DNA. The nucleic acid concentration and purity were assessed using a Nanodrop 2000 spectrophotometer (Thermo, Waltham, USA).

### 2.5. qPCR Analysis of Viral Load

The concentration and purity of the extracted DNA samples were determined. Qualified samples were subjected to qPCR assay to quantify viral load. Following qPCR amplification using primers previously designed in our laboratory [29], each sample was measured in triplicate. The reaction system was prepared according to the TB Green Premix Dimer Eraser (2×) instructions (Takara, Dalian, China): 10 μL of TB Green Premix Dimer Eraser (2×), 0.6 μL each of forward and reverse primers, 1 μL of template, and 7.8 μL of ddH_2_O. The total reaction volume was 20 μL. The reaction conditions were 95°C for 30 s, followed by 40 cycles of 95°C for 5 s, 55°C for 30 s, and 72°C for 30 s. A melting stage was finally performed: 95°C for 1 s, 65°C for 15 s, and 95°C for 1 s. SPF chicken and duck liver tissue samples without viral inoculation were used as negative controls. After qPCR amplification, the obtained Ct values were used to calculate the viral copy number by substituting them into the standard curve. All data analysis and graphical representations for the experiment were performed using Prism 8 software (GraphPad Software, Inc., San Diego, CA, USA).

### 2.6. DAdV‐3 Pathogenicity Assay

Twenty‐one‐day‐old Muscovy ducklings were randomly divided into the challenge group and the control group, with five ducklings in each group. Each Muscovy duckling in the challenge group was intramuscularly injected with 3.56 × 10^4^ TCID_50_ DAdV‐3 solution, while the control group was injected with an equal volume of PBS. After the challenge, they were reared in separate isolators with free access to food and water, maintained at a humidity of 40–60%, and kept under consistent rearing conditions. For 14 consecutive days postchallenge, clinical symptoms of each duck were observed, mortality in each group was recorded, and the time and number of deaths among infected ducks were tallied. Upon reaching a moribund state, ducks were necropsied to observe pathological changes in various tissues and organs.

### 2.7. Tissue Viral Load

Heart, liver, spleen, lung, kidney, thymus, and bursa tissues of Muscovy ducklings were collected from 21‐day‐old Muscovy ducklings infected with DAdV‐3 at 72 hpi, as well as from normal control ducks. Each tissue sample (0.1 g) was mixed with 1 mL PBS buffer and thoroughly homogenized in a low‐temperature tissue homogenizer (Sakezi, Anhui, China) at 10°C and 50 Hz for 80 s. DNA extracted from the tissue samples was used as a template to detect the viral load. All data analysis and graphical representations for the experiment were performed using Prism 8 software (GraphPad Software, Inc., San Diego, CA, USA).

### 2.8. Histopathological Observation

The liver and kidney tissues were fixed in 4% paraformaldehyde (Sangon Biotech, Shanghai, China) at 4°C for 48 h, followed by embedding in paraffin to prepare ultrathin sections. The sections were dewaxed by soaking in xylene for 10 min. Hematoxylin and eosin (HE) staining was performed for 10 min. The stained sections were dehydrated through a graded series of ethanol, soaked in xylene for 2 min to clear the sections, and then sealed with neutral gum on the slides. After drying, pathological changes in the liver and kidney tissues after DAdV‐3 challenge were observed under a light microscope.

### 2.9. Ferroptosis‐Related Factor Analysis

Liver tissues were collected from Muscovy ducks in both the control and challenge groups, with 0.1 g of tissues transferred into 2 mL tubes. Samples were homogenized for 80 s at 50 Hz under 10°C. Following homogenization, samples were centrifuged at 7000 × g for 15 min at 4°C using a Centrifuge 5430R (Eppendorf, Hamburg, Germany). A total of 500 μL supernatant was transferred into a new RNase‐free tubes, mixed thoroughly with 100 μL chloroform, and incubated at room temperature for 2 min, followed by centrifugation at 12,000 × g for 10 min. Then, 200 μL of the supernatant was loaded into columns for RNA extraction using the BioFlux automated nucleic acid extraction and purification system (Hangzhou Bioer Technology Co., Ltd., Zhejiang, China). The purity of isolated RNA samples was determined using a NanoDrop 2000 spectrophotometer. Qualified RNA samples were reverse‐transcribed into cDNA using the PrimeScript RT Reagent Kit with gDNA Eraser (Takara, Dalian, China). Using cDNA as the template and β‐actin as the reference gene, RT‐qPCR was performed to detect the mRNA expression levels of ferroptosis‐related genes, including *SLC7A11*, *FTH1*, *GPX4*, *FANCD2*, *TP53*, and *CHAC1* in Muscovy ducks at 24, 48, and 72 h post‐DAdV‐3 infection. Primers for the detected ferroptosis‐related genes are listed in Table 1. The reaction system was prepared according to the TB Green Premix Ex Taq instructions (Takara, Dalian, China): 12.5 μL of TB Green Premix Ex Taq, 1 μL each of forward and reverse primers, 1 μL of template, and 9.5 μL of ddH_2_O. The total reaction volume was 25 μL. The reaction conditions were 95°C for 30 s, followed by 40 cycles of 95°C for 5 s, 60°C for 30 s, and 72°C for 30 s; 95°C for 1 s, 65°C for 15 s, and 95°C for 1 s. Normal duck liver tissue samples were used as negative controls. Each sample was measured in triplicate. The relative mRNA expression levels were calculated using the 2^-ΔΔCT^ method. All data analysis and graphical representations for the experiment were performed using Prism 8 software (GraphPad Software, Inc., San Diego, CA, USA). The glutathione (GSH) content in liver tissues was subsequently determined at 0, 24, 48, and 72 h post‐DAdV‐3 infection according to the manufacturer’s instructions of the assay kit (Nanjing Jiancheng Bioengineering Institute, Cat. Number A061‐1‐2, Nanjing, China).

**Table 1:** The primer sequences of ferroptosis‐related genes.

Gene name	Primer sequences (5′→ 3′)	Product sizes/bp
*SLC7A11*	F‐TCTGGCGGTCACTACACCTA R‐TACCGTCCAAATGCCAACGA	128
*FTH1*	F‐TACTTCCTGCACCAGTCCCA R‐GTCAGCCCATTCTCCCAGTC	134
*GPX4*	F‐GAGGGCCAAGTGGTGAAGAG R‐GGCGAGATGAGCAGGTCTAC	94
*TP53*	F‐TACCACGACGACGAGACCAC R‐GTGATGATGGCGAGGATGGG	155
*CHAC1*	F‐ACAAGGATGCAGAGGAACCC R‐CCGCTTGAGGCCATGATTTG	118
*β-Actin*	F‐GGTATCGGCAGCAGTCTTA R‐TTCACAGAGGCGAGTAACTT	158

### 2.10. Gene Characteristic Analysis

Viral DNA was extracted from the supernatant of duck kidney cells infected with DAdV‐3 using a viral DNA extraction kit. The complete genome of DAdV‐3 was obtained through high‐throughput sequencing technology. Quality assessment and quality control of the Illumina and PacBio sequencing were conducted, followed by the assembly of the genome sequence and analysis of its genetic elements.

### 2.11. Phylogenetic Analysis

Phylogenetic analysis was conducted using SnapGene, DNAstar 5.0, and MEGA 11 software, focusing on the complete genome sequence and key gene sequences, including DNA polymerase, Hexon, Fiber1, and Fiber2 genes. This analysis was compared with the nucleotide sequence identity of all DAdV‐3 (DAdV‐B and DAdV‐2) strains available in GenBank. Notably, FJGT01 (GenBank ID: MH777395), GDMM10 (GenBank ID: MH777398), AHAQ13 (GenBank ID: MH777396), ZJJH (GenBank ID: MH777397), FJFZ‐2024 (GenBank ID: PZ263083), and FJNP‐2021 strains from 2015 to 2024 were isolated and preserved in our avian disease laboratory.

## 3. Results and Analysis

### 3.1. DAdV‐3 Strain Culture

DEK cells grow to a fresh monolayer after 24 h of in vitro culture and were subsequently inoculated with DAdV‐3 viral suspension. Significant cytopathic effects were observed at 48 hpi, primarily manifested as cell swelling and aggregation (Figure 1A). With the prolongation of DAdV‐3 infection, some cells detach and become semisuspended state. In contrast, control group cells exhibited normal growth.

**Figure 1:**
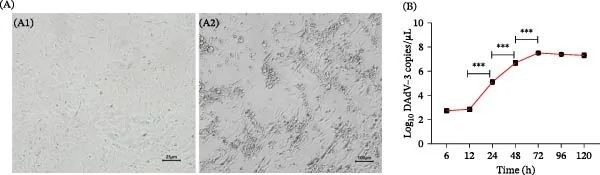
DAdV‐3 infection in primary duck embryo kidney (DEK) cells. (A) Cytopathic effects infected by DAdV‐3 FJNP‐2021 strain in DEK cells. (A1) Normal primary DEK cells. (A2) Cytopathic effect of DAdV‐3 infection in primary DEK cells at 48 hpi. (B) Virus proliferation curve of DAdV‐3 FJNP‐2021 strain in DEK cells postinfection ( ^∗∗∗^ *p* < 0.001).

### 3.2. Viral Proliferation Dynamics

The qPCR assay was used to quantify the viral nucleic acid levels of DAdV‐3 in DEK cells at 6, 12, 24, 48, 72, 96, and 120 hpi. As illustrated in Figure 1B, DAdV‐3 exhibited rapid proliferation in primary DEK cells between 12 and 72 hpi, with highly significant differences observed at 24, 48, and 72 hpi (*p* < 0.001). Subsequently, the viral copy numbers stabilized between 72 h and 120 hpi, indicating a plateau phase of viral proliferation.

### 3.3. Ultrastructural Analysis of DAdV‐3–Infected DEK Cells

At 48 hpi of DEK cells with DAdV‐3, viral assembly occurs within the nucleus of the DEK cells (Figure 2A). Individual cell cross‐sections reveal the presence of viral assembly factories, where viral particles are tightly arranged in a crystalline lattice, with a diameter of ~70 nm (Figure 2B,C: Green arrows indicate viral particles). Additionally, damaged and deformed mitochondria were observed in the cytoplasm, with some mitochondria appearing smaller in size and exhibiting a partial loss of cristae (indicated by yellow arrows in Figure 2D). Furthermore, autophagosome‐like vesicular structures encapsulating damaged organelles were also observed in the cytoplasm, as indicated by the black arrows in Figure 2A,C and D.

**Figure 2:**
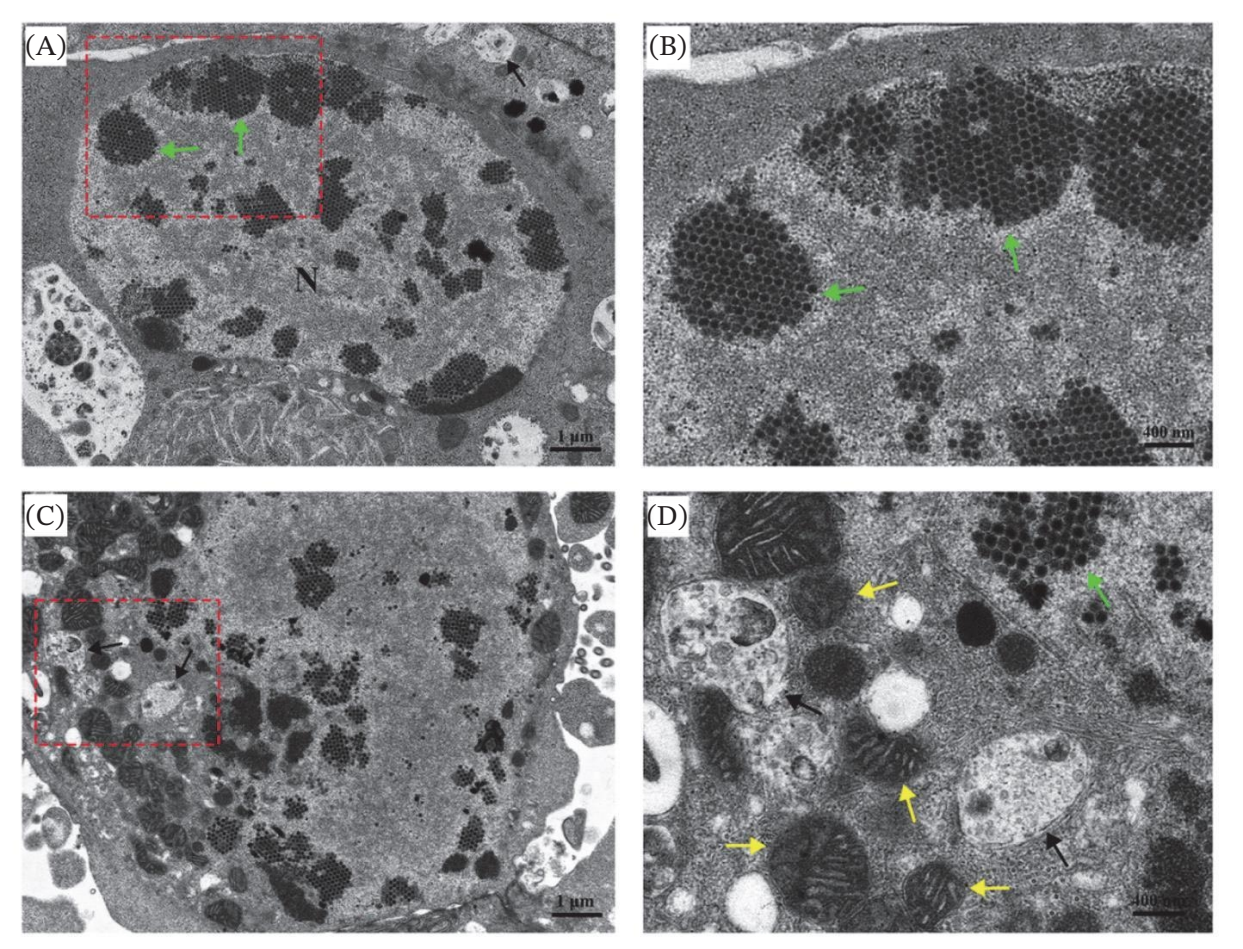
Ultrastructural changes in DEK cells infected with DAdV‐3. (A) DEK cells were infected by DAdV‐3 FJNP‐2021 strain. (B) An enlarged view of the area outlined by the red dashed box in (A). (C) Autophagy in DEK cells infected with DAdV‐3. (D) An enlarged view of the area outlined by the red dashed box in (C). Viral particles (green arrows), mitochondria (yellow arrows), and vesicle‐like autophagosomal structures (black arrows).

### 3.4. Pathogenicity Analysis of DAdV‐3 Strain

#### 3.4.1. Clinical Symptoms and Necropsy Lesions

Following the challenge, the experimental group of Muscovy ducklings exhibited symptoms indicative of depression and a reduced feed intake. Notably, some ducks displayed signs of leg weakness and frequent crouching ~3–4 days postinfection. By the third day postinfection, mortality was observed among the Muscovy ducklings. In contrast, the control group exhibited no significant abnormal symptoms throughout the duration of the challenge assay. Necropsy findings of deceased and moribund ducks from the challenge group revealed enlarged and hemorrhagic livers, characterized by necrotic foci in localized regions. Additionally, hemorrhagic spots were observed in certain areas of the kidneys, along with a small accumulation of effusion in the pericardial cavity (Figure 3A).

**Figure 3:**
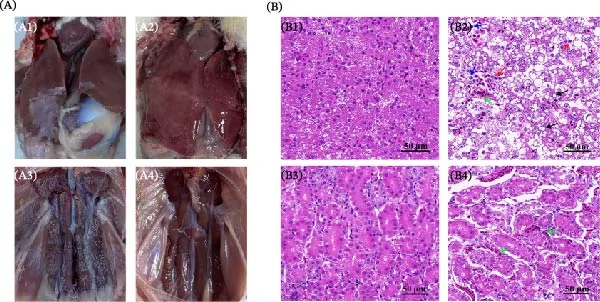
DAdV‐3 infection induces pathological lesions in the liver and kidney of Muscovy ducklings. (A) Postmortem lesions of Muscovy ducklings infected with DAdV‐3 strain. (A1) Normal liver. (A2) Liver lesions after DAdV‐3 infection. (A3) Normal kidney. (A4) Kidney lesions after DAdV‐3 infection. (B) The histopathological changes of Muscovy ducklings infected with DAdV‐3 strain. (B1) Normal liver tissue. (B2) Liver tissue after DAdV‐3 infection. (B3) Normal kidney tissue. (B4) Kidney tissue after DAdV‐3 infection. Red blood cell infiltration (green arrow), basophilic inclusion bodies (blue arrow), nuclear enlargement (red arrow); numerous round, variably sized, well‐demarcated vacuoles in the cytoplasm of necrotic hepatocytes (black arrow).

#### 3.4.2. Histopathological Changes

Following infection with DAdV‐3, significant pathological alterations were observed in the kidney and liver tissues of Muscovy ducklings. HE staining revealed severe pathological lesions in liver tissues following DAdV‐3 infection. Extensive hepatocellular degeneration and necrosis were observed, characterized by numerous round, variably sized, and well‐demarcated vacuoles in the cytoplasm of necrotic hepatocytes, indicating marked steatosis. Additionally, some hepatocytes displayed enlarged nuclei with basophilic intranuclear inclusion bodies were identified. Furthermore, erythrocyte congestion was evident in the hepatic sinusoids, accompanied by mild infiltration of inflammatory cells surrounding the necrotic foci (Figure 3B). The structural integrity of the kidney tissue postinfection was disrupted, with erythrocyte exudation observed in the interstitium. The renal tubular epithelial cells displayed signs of swelling, degeneration, and necrosis. Furthermore, several renal tubules exhibited narrowed or occluded lumina, which contained sloughed epithelial cell debris along with mild infiltration of inflammatory cells (Figure 3B).

### 3.5. Distribution of Viral Load in Different Organs

DAdV‐3 was identified via PCR assay across multiple tissues, including the heart, liver, spleen, lungs, kidneys, thymus, and bursa of infected ducks (Figure 4A), indicating a broad tissue tropism. The distribution of viral load across various organs in Muscovy ducklings following DAdV‐3 infection was assessed using qPCR, as shown in Figure 4B. Three days postinfection with the FJNP‐2021 strain, the viral loads in different tissues were ranked from highest to lowest: liver, spleen, lung, heart, kidney, bursa, and thymus. The liver tissue exhibited the highest viral load at 1.26 × 10^6^ copies/mg, while the kidney demonstrated a lower viral load of 3.98 × 10^3^ copies/mg. The immune organ, the spleen, recorded a viral load of 3.16 × 10^5^ copies/mg; the bursa had 1.26 × 10^3^ copies/mg; and the thymus had 3.16 × 10^2^ copies/mg. Moreover, the viral load in the liver was significantly higher than that in the spleen, with a highly significant difference (*p* < 0.01). The viral load of the liver was markedly higher than that of the kidney, showing an extremely significant statistical difference (*p* < 0.001).

**Figure 4:**
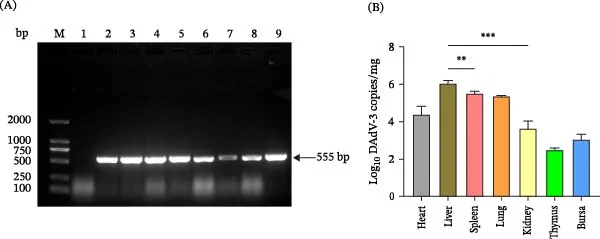
Detection of DAdV‐3 in different tissues postinfection. (A) PCR analysis of DAdV‐3 distribution in tissues of Muscovy ducks. M, DL2000 DNA Marker; Lane 1, negative control; Lane 2, heart; Lane 3, liver; Lane 4, spleen; Lane 5, lung; Lane 6, kidney; Lane 7, thymus; Lane 8, bursa; Lane 9, positive control. (B) Detection of viral loads in different tissues via qPCR assay ( ^∗∗^ *p* < 0.01,  ^∗∗∗^ *p* < 0.001).

### 3.6. Ultrastructural Analysis of DAdV‐3–Infected Hepatocytes

Typical pathological lesions were observed at the ultrastructural level in the liver hepatocytes of DAdV‐3–infected Muscovy ducklings (Figure 5). Further observations indicate a significant accumulation of lipid droplets in liver hepatocytes following DAdV‐3 infection. Additionally, some mitochondria appeared smaller in size with reduced or absent cristae, accompanied by disordered endoplasmic reticulum (ER) structures surrounding the mitochondria (Figure 5A–D: Blue arrows indicate lipid droplets, red arrows indicate the ER, green arrows indicate viral particles, and yellow arrows indicate mitochondria).

**Figure 5:**
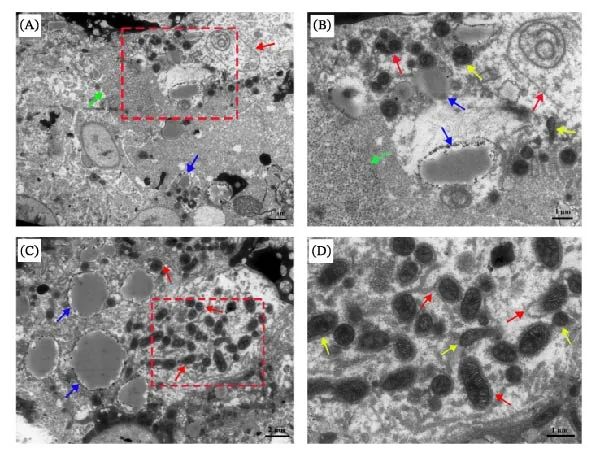
Ultrastructural changes in hepatocytes of Muscovy ducklings infected with DAdV‐3. (A) Ultrastructural morphology of hepatocytes in Muscovy ducklings infected with DAdV‐3 FJNP‐2021 strain. (B) An enlarged view of the area outlined by the red dashed box in (A). (C) Lipid droplets and disordered endoplasmic reticulum surrounding the mitochondria in the cytoplasm of injured liver cells. (D) An enlarged view of the area outlined by the red dashed box in (A). Viral particles (green arrows), lipid droplets (blue arrows), endoplasmic reticulum (red arrows), and mitochondria (yellow arrows).

### 3.7. Ferroptosis‐Related Factor Analysis

To investigate whether DAdV‐3 infection triggers ferroptosis in hepatocytes, we analyzed the mRNA expression levels of key ferroptosis‐related genes (*SLC7A11*, *FTH1*, *GPX4*, *TP53*, and *CHAC1*) at 24, 48, and 72 hpi. The results showed that the mRNA expression levels of the ferroptosis suppressor genes *SLC7A11*, *FTH1*, and *GPX4* progressively decreased over time (Figure 6A–C). Notably, the *SLC7A11* expression level was consistently downregulated at 24, 48, and 72 h, with reductions of 2.15‐fold, 3.17‐fold, and 4.76‐fold, respectively. In contrast, the transcriptional levels of the proferroptotic genes *TP53* and *CHAC1* were significantly upregulated (Figure 6D,E). *TP53* mRNA expression level remained relatively stable at 24 and 48 h but increased by 2.82‐fold at 72 h. *CHAC1* mRNA expression showed no obvious change at 24 h but was significantly upregulated at 48 and 72 h, with increases of 4.82‐fold and 4.93‐fold, respectively. Collectively, this differential expression pattern of ferroptosis‐related genes was characterized by the downregulation of ferroptosis suppressor genes (*SLC7A11*, *GPX4*, and *FTH1*) and the upregulation of proferroptotic genes such as *TP53* and *CHAC1*. Besides, the GSH content in liver tissues was measured at 0, 24, 48, and 72 hpi with DAdV‐3. As illustrated in Figure 6F, the GSH levels of liver tissues were significantly reduced at 24, 48, and 72 hpi compared to the control group (*p* < 0.001), suggesting that DAdV‐3 infection induced GSH depletion in liver tissues. These results indicated that DAdV‐3 infection may induce ferroptosis in hepatocytes by inhibiting the SLC7A11–GPX4 antioxidant axis while simultaneously activating proferroptotic signals (*TP53* and *CHAC1*).

**Figure 6:**
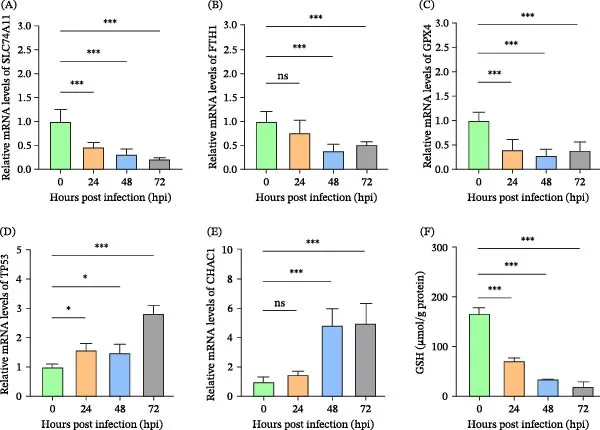
mRNA expression levels of ferroptosis‐related factors in liver hepatocytes of Muscovy ducklings infected with DAdV‐3 strain. (A) *SLC7A11*; (B) *FTH1*; (C) *GPX4*; (D) *TP53*; (E) *CHAC1*; (F) the GSH content in liver tissues was measured at 0, 24, 48, and 72 h postinfection with DAdV‐3 (ns, *p* > 0.05;  ^∗^ *p* < 0.05;  ^∗∗∗^ *p* < 0.001).

### 3.8. Genetic Characterization Analysis

The complete genome sequence of the FJNP‐2021 strain was obtained using Illumina sequencing. The genome of the FJNP‐2021 strain is 43,842 bp in length, with a GC content of 47%, and contains 26 open reading frames (Figure 7). The FJNP‐2021 strain has been submitted to GenBank and assigned the Accession Number PZ263084.

**Figure 7:**
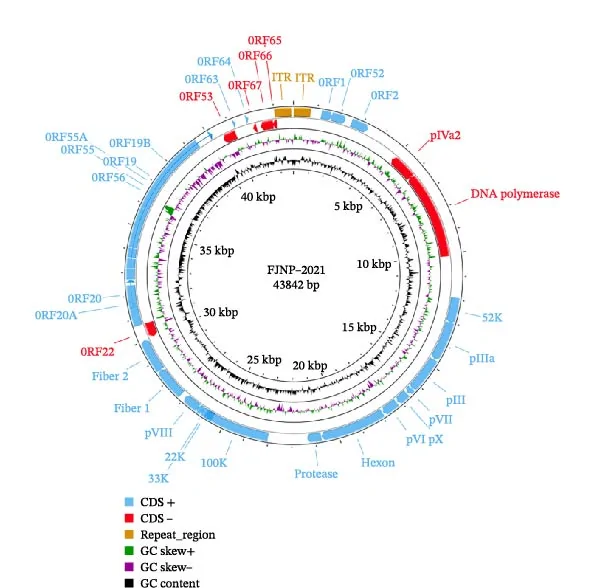
Circular genome map of FJNP‐2021 strain.

### 3.9. Phylogenetic Analysis of Complete Genome

A total of 33 DAdV‐3 (DAdV‐B) strains are currently deposited in GenBank, all of which were isolated in China. The phylogenetic tree, constructed from the complete nucleotide sequences of the FJNP‐2021 strain and the FJFZ‐2024 strain (GenBank ID: PZ263083), both isolated in our laboratory, along with the HB20 strain from Hubei province, the GTL strain, the BGMH strain, and the FJGT01 strain from Fujian province, as well as the HF‐AH‐2022 strain isolated in Anhui province, CHGDLKQ202002 and GDMM10 from Guangdong province, the representative strain CH‐GD‐12‐2014, and other domestic DAdV‐3 strains, showed that all clustered on the same lineage, indicating close genetic relationships of Chinese DAdV‐3 strains (Figure 8). The nucleotide sequence identity between the FJNP‐2021 strain and domestic strains such as HF‐AH‐2022, GTL, BGMH, GDMM10, CHGDLKQ202002, CH‐GD‐12‐2014, and HB20 ranged from 99.8% to 99.9%. Specifically, the FJNP‐2021 strain shares 99.9% nucleotide identity with the Guangdong representative strain CH‐GD‐12‐2014 and the Fujian isolates FJFZ‐2024, FJZZ, FJPT20161124, CH‐FJYHJ‐201805, CH‐FJLYE‐201805, FJGT01, CH‐FJLKK‐201804, CH‐FJCKL‐201804, GTL, and BGMH. In comparison, the FJNP‐2021 strain exhibits 92.3% nucleotide identity with the French representative GR strain, indicating that they belong to different evolutionary lineages. In contrast, the identity with DAdV‐1 is only 40.9%, while the identity with DAdV‐4 is 55.0% (Figure 6).

**Figure 8:**
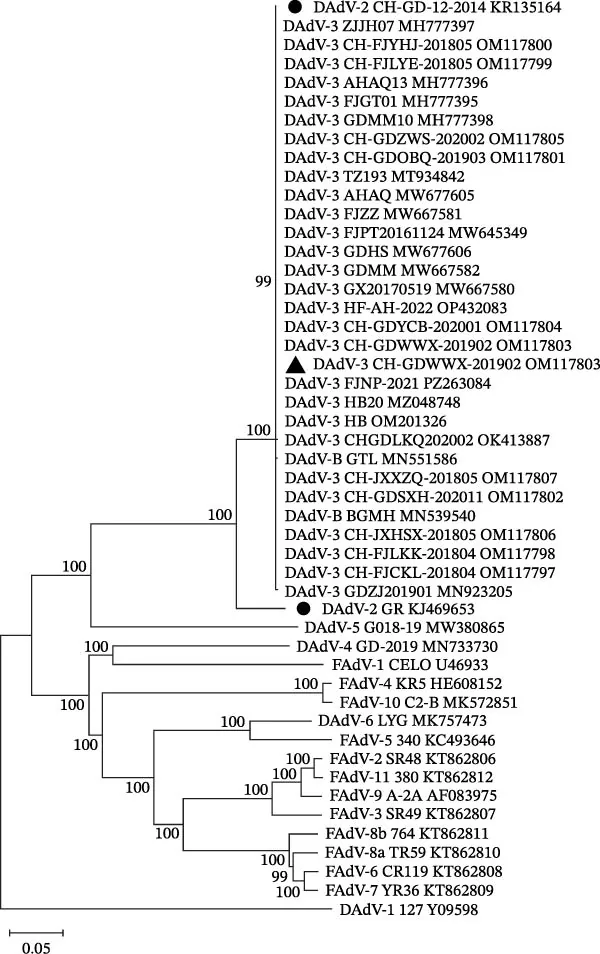
Phylogenetic analysis of complete genome sequences of DAdV‐3 strains available in GenBank from 2014 to 2024. The scale bar represents the distance unit between sequence pairs. Representative strains GR and CH‐GD‐12‐2014 are indicated by black dot, and the FJNP‐2021 strain is marked with black triangle.

### 3.10. Phylogenetic Analysis of DNA Polymerase Genes

The adenovirus DNA polymerase belongs to the DNA polymerase α family, a key enzyme in viral DNA replication, whose structural and functional features are similar to DNA polymerases from other DNA viruses, such as herpesviruses and poxviruses [30, 31]. Research on FAdVs has revealed that variations in the DNA polymerase gene and genetic distance are essential for the classification of adenoviruses. The DNA polymerase protein directly influences the replication capability of viral strains. The length of the DNA polymerase gene is 4026 bp. Phylogenetic analysis reveals that the DNA polymerase gene of the FJNP‐2021 strain exhibits a close genetic relationship with CH‐GD‐12‐2014 and CH‐GDLKQ‐202002 strains from Guangdong province, the HB20 strain from Hubei province, and the BGMH and GTL strains from Fujian province, all of which cluster within the major lineage with 100% identity (Figure 9). In contrast, the FJNP‐2021 strain and the French representative strain GR form a distinct minor lineage, characterized by a greater evolutionary distance and 99.8% identity. Notably, FJNP‐2021 and FJFZ‐2024 strains exhibited 49.8% nucleotide identity with DAdV‐1 (EDSV) and 58.7% nucleotide identity with DAdV‐4.

**Figure 9:**
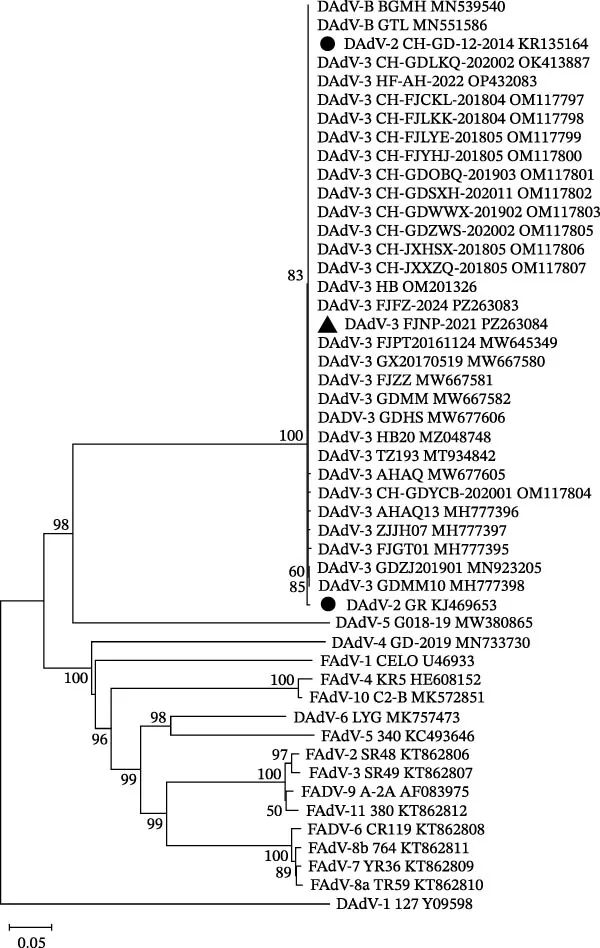
Phylogenetic analysis of DNA polymerase genes. Representative strains GR and CH‐GD‐12‐2014 are indicated by black dot, and the FJNP‐2021 strain is marked with black triangle.

### 3.11. Phylogenetic Analysis of Hexon Genes

Phylogenetic analysis of the Hexon gene indicates that the FJNP‐2021 strain is most closely related to the representative strain CH‐GD‐12‐2014 from Guangdong province in China. Additionally, the FJNP‐2021 strain shares a close relationship with DAdV‐3 (DAdV‐B) strains from various regions in China, including Fujian, Shanghai, Hubei, Anhui, and Guangdong, all of which belong to the major evolutionary lineage, exhibiting 100% nucleotide sequence identity (Figure 10). Furthermore, FJNP‐2021 demonstrates a 100% nucleotide sequence identity with FJFZ‐2024. In contrast, FJNP‐2021 diverges from the representative strain GR, isolated in France, showing a lower identity of 76.6%. The nucleotide sequence identity with DAdV‐1 is 54.4%, with DAdV‐4 is 66.2%, and with duck‐origin FAdV‐4 (GenBank ID: ON929900) is 65.71%.

**Figure 10:**
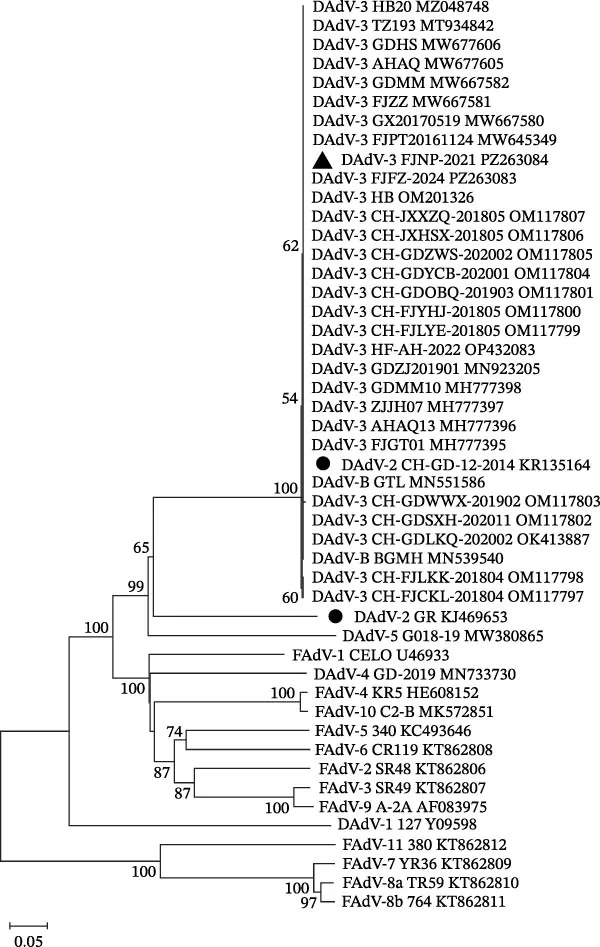
Phylogenetic analysis of Hexon genes. Representative strains GR and CH‐GD‐12‐2014 are indicated by black dot, and the FJNP‐2021 strain is marked with black triangle.

### 3.12. Phylogenetic Analysis of Fiber Genes

The Fiber1 gene of the FJNP‐2021 strain, isolated in this study, clustered within the same lineage as Chinese isolates of DAdV‐3 strains, including the Guangdong representative strain CH‐GD‐12‐2014, HF‐AH‐2022 from Anhui province, FJGT01 strains from Fujian province, and HB20 from Hubei province. Additionally, the Fiber1 gene of the FJNP‐2021 strain clustered with domestic DAdV‐3 strains such as CHGDLKQ202002 and GDMM10 strains from Guangdong province; HB20 strain from Hubei province; and FJPT20161124, FJGT01, and FJFZ‐2024 strains from Fujian province, exhibiting nucleotide homologies ranging from 99.9% to 100%. Notably, the Fiber1 genes of GTL, BGMH, CH‐FJLKK‐201804 and CH‐FJCKL‐201804 strains from Fujian province formed a distinct minor subclade together with CH‐JXXZQ‐ 201805 and CH‐JXHSX‐201805 strains from Jiangxi province, CH‐GDSXH‐202011 strain from Guangdong province. In contrast, the FJNP‐2021 strain was located in a different subclade from these seven strains and shared 98.3%–98.8% nucleotide identity with them. These results indicated the presence of genetic diversity of Fiber1 genes among Chinese isolates of DAdV‐3 strains. The Fiber1 gene of the FJNP‐2021 strain was positioned on a separate lineage from the French representative GR strain, sharing only a 35.2% nucleotide identity. Furthermore, the FJNP‐2021 strain shared 31.7% identity with the G018‐19 strain, 34.6% with the GD‐2019 strain, and 32.3% with the DAdV‐1 strain (Figure 11A).

**Figure 11:**
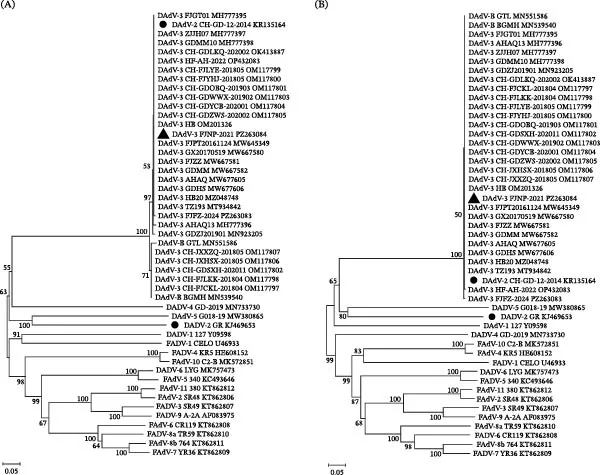
Phylogenetic analysis of Fiber1 and Fiber2 genes. (A) Fiber1 genes. (B) Fiber2 genes. Representative GR strain and CH‐GD‐12‐2014 strain are indicated by black dot, and the FJNP‐2021 strain is marked with black triangle.

The Fiber2 gene of the FJNP‐2021 strain clustered within the same lineage as Chinese isolates of DAdV‐3 strains, including Guangdong representative strain CH‐GD‐12‐2014, as well as several Fujian strains (FJGT01, GTL, BGMH, FJPT20161124, and FJFZ‐2024), GDMM10 and CHGDLKQ202002 from Guangdong province, HB20 from Hubei province, and HF‐AH‐2022 from Anhui province, exhibiting nucleotide sequence identity ranging from 99.9% to 100%. In contrast, the Fiber2 gene of the FJNP‐2021 strain and the French representative strain GR share a nucleotide identity of 51%, with the GR strain forming a distinct minor lineage. The identity of the Fiber2 gene of the FJNP‐2021 strain with the G018‐19 strain is 40.3%, with the GD‐2019 strain is 38.8%, and with the DAdV‐1 strain is 37.6% (Figure 11B).

## 4. Discussion

In recent years, DAdV‐3 has emerged as a spreading pathogen, with clinical cases reported in Muscovy duck farms across multiple provinces in China, including Fujian, Guangdong, Zhejiang, and Anhui [32]. While the virus primarily affects Muscovy ducklings [12, 24], it causes occasional infections in shelducks; for instance, a DAdV‐3 strain was isolated from shelducks by Shi et al. [22] in 2019. Clinically, DAdV‐3 infection in Muscovy ducks is characterized by swelling and hemorrhage of the liver and kidneys. DAdV‐3 is frequently coinfected with MDPV, DHBV, and AAV [24, 33]. DAdV‐3 infections occur sporadically, primarily affecting Muscovy ducklings, particularly during coinfections that exacerbate morbidity and mortality. The increasing incidence of DAdV‐3 infections has resulted in significant economic losses within the waterfowl industry in China.

Following experimental challenge, DAdV‐3 was detected in the heart, liver, spleen, lungs, kidneys, thymus, and bursa, indicating a broad tissue tropism in infected Muscovy ducklings, consistent with the findings reported by Cao et al. [32]. Interestingly, analysis of viral load in various organs post‐DAdV‐3 infection revealed that the liver is a significant target organ, with the viral load in the spleen being second only to that in the liver. This hepatotropic and lymphoid predilection is consistent with the established biology of *Aviadenoviruses*, which typically establish infection in the liver and lymphoid tissues and induce inclusion‐body hepatitis [34]. Although DAdV‐3 primarily infects Muscovy ducklings and is characterized by a low lethality rate as a typically subclinical pathogen, the relatively high detection rate of DAdV‐3 on farms indicates a significant prevalence of viral carriage. This viral carriage can lead to secondary and coinfections, thereby exacerbating clinical symptoms.

This study provides the first ultrastructural evidence of DAdV‐3‐induced liver injury in ducklings. The characteristic lesions included extensive lipid droplet formation, disorganized ER structures, and some mitochondria that appeared smaller in size with reduced or absent cristae, which were closely surrounded by the ER in DAdV‐3–infected hepatocytes. Previous studies have shown that infection with certain viruses, such as flavivirus and infectious bursal disease virus, can induce cytopathological changes in host cells, including lipid droplet accumulation, ER stress (ERS), and subsequent structural disruption [35, 36]. Similar morphological features have also been observed in FAdV‐4–infected host cells, a FAdV belonging to different serotypes [37–39]. In this study, we observed ferroptosis‐like morphological features in the hepatic ultrastructure of virus‐infected ducklings. Specifically, DAdV‐3 infection resulted in some mitochondrial shrinkage and reduced or absent cristae in hepatocytes, as well as extensive wrapping of mitochondria by the ER. Previous studies have demonstrated that ER–mitochondria contact sites serve as “initiation hubs” for phospholipid peroxidation, allowing lipid peroxides to spread from the ER to mitochondria, thereby triggering mitochondrial dysfunction, fission, and ultimately promoting ferroptosis [40]. Furthermore, our results indicate that DAdV‐3 infection significantly downregulated the ferroptosis‐suppressive genes *SLC7A11*, *GPX4*, and *FTH1* expression levels while upregulating the proferroptotic genes *TP53* and *CHAC1* expression levels, collectively exacerbating ferroptosis. It has been established that the upregulation of *TP53* suppresses *SLC7A11* transcription, which reduces cystine uptake and GSH synthesis [41], accompanied by GPX4 downregulation. Additionally, the upregulation of *CHAC1* aggravates ferroptosis and oxidative stress injury by depleting GSH and inhibiting GPX4 expression [42]. Moreover, the deficiency of *FTH1* induces ferritinophagy and promotes ferroptosis [43]. Taken together, our findings suggest that DAdV‐3 infection induces liver injury in ducklings and may activate the p53–SLC7A11–GPX4 pathway in hepatocytes postinfection. Combined with the typical ultrastructural pathological features, our study preliminarily demonstrates that DAdV‐3 infection could induce hepatic lipid metabolism disorder and potentially trigger ferroptosis, thereby exacerbating hepatocyte injury. However, the pathogenic mechanism underlying liver injury remains to be further investigated.

This study obtained the complete genome sequence of DAdV‐3 utilizing high‐throughput sequencing technology. Phylogenetic analysis of the complete genome sequence revealed that the FJNP‐2021 strain clusters in the same major lineage with other domestic Muscovy duck DAdV‐3 isolates, indicating a high degree of genetic conservation and confirming that the FJNP‐2021 strain represents a typical member of the dominant DAdV‐3 lineage currently circulating in China. This conservation was reinforced by gene‐by‐gene analyses. The DNA polymerase, Hexon, Fiber1, and Fiber2 all clustered the FJNP‐2021 strain with Chinese DAdV‐3 strains isolated from 2014 to 2024 available in GenBank, supporting the view that the contemporary Chinese epidemic is sustained by a genetically homogenous DAdV‐3 population. Notably, the Fiber1 gene of the DAdV‐3 strains in China exhibited two distinct subclades, suggesting the presence of genetic diversity of Fiber1 genes among Chinese isolates of DAdV‐3 strains. Previous research by Shi et al. [22] revealed genetic differences of the TZ193 strain in the DAdV‐3 genome, particularly in the ORF67 gene, which contains a premature stop codon. The results indicate genomic diversity among the Chinese isolates of DAdV‐3 strains, suggesting that circulating strains may exhibit variations in replication efficiency and virulence potential. Therefore, continuous molecular epidemiological surveillance is essential for monitoring the emergence of variant strains in duck flocks. While the present study provides a comprehensive genomic and phylogenetic characterization of the Chinese DAdV‐3 isolates, the functional roles of key genes, particularly the amino acid residue mutations in the Fiber1 gene, require experimental verification in future studies, such as through reverse genetics or site‐directed mutagenesis.

## 5. Conclusion

Taken together, this study investigated the biological characteristics of DAdV‐3 in DEK cells and its pathogenicity in Muscovy ducklings. Our in vivo results demonstrated that the DAdV‐3 infection induced severe hepatitis in the Muscovy ducklings. Additionally, we preliminarily explored the genomic characteristics of the FJNP‐2021 strain and other DAdV‐3 (DAdV‐2) strains available in GenBank. The Chinese isolates of DAdV‐3 strains originate from the same lineage and are generally evolutionarily conserved. Notably, the Fiber1 genes of these isolates form two independent subclades. These genomic features are essential for enhancing our understanding of the genetic diversity and epidemiology of DAdV‐3 strains in China. This research establishes a foundational basis for future in‐depth investigations into the pathogenic mechanisms of DAdV‐3 and the development of effective control strategies against DAdV‐3.

## Author Contributions

**Chunhua Zhu**: conceptualization, methodology, project administration, formal analysis, writing – original draft, review and editing. **Zhiwang Tang**: methodology, software, formal analysis. **Pei Yang**: methodology, formal analysis, data curation, software. **Longfei Chen, Guanghua Fu, Qizhang Liang, Zhen Chen, and Cuiteng Chen**: methodology. **Yu Huang**: review and editing. **Chunhe Wan**: funding acquisition, review and editing.

## Funding

This work was supported by the Fujian Provincial Public Research Institute of Fundamental Research (Grants 2025R1071 and 2025R1024009), the China Agriculture Research System (Grant CARS‐41‐18), and the Research and Technology Program of Fujian Academy of Agricultural Sciences (Grants YCZX202412 and DWHZ2026‐04).

## Disclosure

All the authors have contributed to the manuscript and approved the submitted version.

## Ethics Statement

The Muscovy duckling challenge experiments involved in this study received approval from the Ethics Review Committee of the Institute of Animal Husbandry and Veterinary Medicine, Fujian Academy of Agricultural Sciences (Animal Ethics Approval Number 202402FJ016).

## Conflicts of Interest

The authors declare no conflicts of interest.

## Data Availability

The data that support the findings of this study are available upon reasonable request. The data are not publicly available due to ethical considerations.
